# Sociodemographic influences on youth sport participation and physical activity among children living within concentrated Hispanic/Latino rural communities

**DOI:** 10.3389/fpubh.2024.1345635

**Published:** 2024-02-21

**Authors:** Mary J. Von Seggern, Ann E. Rogers, Michaela A. Schenkelberg, Debra K. Kellstedt, Gregory J. Welk, Robin High, David A. Dzewaltowski

**Affiliations:** ^1^Department of Health Promotion, College of Public Health, University of Nebraska Medical Center, Omaha, NE, United States; ^2^School of Health and Kinesiology, College of Education, Health, and Human Sciences, University of Nebraska at Omaha, Omaha, NE, United States; ^3^Texas A&M AgriLife Extension, Family and Community Health, Texas A&M University, College Station, TX, United States; ^4^Department of Kinesiology, College of Human Sciences, Iowa State University, Ames, IA, United States; ^5^Department of Biostatistics, College of Public Health, University of Nebraska Medical Center, Omaha, NE, United States

**Keywords:** child/children, physical activity, rural, youth sport, Hispanic/Latino, health disparities

## Abstract

**Introduction:**

Lack of physical activity (PA) among children living in rural communities is a documented public health problem. Although studies have examined community conditions defined by a rural–urban dichotomy, few have investigated rural community conditions with a concentration of Hispanic/Latino people. This cross-sectional study examined sociodemographic characteristics associated with youth sport (YS) participation and daily PA among children living within concentrated Hispanic/Latino rural U.S. Midwest communities.

**Methods:**

During spring 2022, 97% of 3rd–6th grade children (*n* = 281, aged approximately 8–12 years) attending school in rural Midwestern communities (*n* = 2) with >50% concentration of Hispanic students participated in the Wellscapes Project, a community randomized trial. Participants completed the Youth Activity Profile and supplemental National Survey of Children’s Health questions assessing PA behaviors and YS participation. Caregivers of a subsample of children (*n =* 215; males, *n* = 93; females, *n* = 122) consented to pair their child’s survey results with school enrollment records (e.g., free/reduced lunch status and race and ethnicity). Mixed models with community as a random effect examined main and interaction effects of grade, sex, ethnoracial status, and family income on YS participation and these sociodemographic characteristics and YS participation on daily moderate-to-vigorous PA (MVPA).

**Results:**

Approximately half of children participated in YS. Non-Hispanic White children (*n* = 82) were over five times more likely to participate in YS than Hispanic peers (*n* = 133) (OR = 5.54, 95% CI = 2.64–11.61, *p* < 0.001). YS participants accumulated 8.3 ± 2.3 more minutes of daily MVPA than non-participants (*p* < 0.001). Sixth graders, females, and Hispanic children reported lower daily MVPA than comparison groups (*p* < 0.05). Significant interaction effects on daily MVPA between grade and ethnoracial status (*F*(3, 204) = 3.04, *p* = 0.030) were also found.

**Discussion:**

Disparities in sport participation and PA outcomes based on sociodemographic characteristics exist among children living in ethnoracially diverse rural communities. Strategies to promote YS participation, including community structural changes, may help reduce PA disparities. The research provides valuable insights for policymakers, public health professionals, and community members to address YS participation barriers, not limited to cost, while considering other PA-promotion efforts to improve child population health.

## Introduction

Although the benefits of physical activity (PA) are well-established (1, 2), over 75% of children and adolescents, aged 6–17 years, living in the United States (U.S.) are failing to meet the current federal PA guidelines (3). As a consequence of this documented public health problem, the fifth iteration of the U.S. Department of Health and Human Services’ Healthy People initiative includes 2030 national objectives targeting an approximate 7% increase in the total proportion of U.S. children and adolescents who participate in the recommended 60 min or more of moderate-to-vigorous PA (MVPA) every day, from 23.6 to 30.4% and 23.2 to 30.6%, respectively (4).

Communities are social systems of interaction that create the structural conditions that influence children’s health behaviors, like PA (5, 6). The structural conditions are labeled the social determinants of health, recognizing conditions in the environments where people live, learn, play, and age also affect PA and other health behaviors and outcomes (4). One way to categorize community conditions is by characterizing communities in a dichotomous or unidimensional fashion, such as the traditional rural–urban dichotomy (e.g., between places inside and outside metro areas) (7). But, studies using this approach to examine children’s PA have produced divergent results, in which some studies have found that rural children are less active than urban children while others have found the opposite to be true (8–12). Scholars contend this classification may not adequately reflect the complexity within communities (7, 13). Specifically, Sandercock et al. argue that the oversimplification of these spatially based concepts, explicitly for under-researched children living in rural communities with diversified contexts, setting environments, and cultural processes, may be one reason for diversity of PA outcomes, and they suggest that racial processes, among others, should not be discounted (14, 15).

Another way to characterize community conditions is to define communities by concentration of ethnoracial diversity. But, studying ethnoracial diversity in isolation does not consider emerging social system processes resulting from the interaction between rural–urban and ethnoracial diversity (7, 13). Although rural communities may be assumed to be homogeneous (e.g., fundamentally conservative, White, and economically dependent on farming), there is documented growing ethnoracial diversity in these communities over the past decade, marked by an influx of Hispanic immigrants into rural communities with service and manufacturing economic opportunities; thus, demanding a shift in the stereotypical rural rhetoric (16–18). Since the late 1970s, when family farms became fewer and larger with the rise of agribusiness, meat-processing plants began consolidating to areas where animals were raised, particularly in rural U.S. communities throughout the Midwest and Great Plains states (e.g., Nebraska, Iowa, and Kansas), to reduce costs (e.g., cheaper land and lower labor and transportation costs) (19). In response to labor shortages, many plants turned to immigrants, the majority of Hispanic origin, to fill this void, while relying on workers’ networks for continual employee recruitment (20). Consequently, the growing diversity of this workforce, coupled with changes in industry, have impacted the sociodemographic and economic characteristics of Midwest rural communities and the settings (e.g., schools) nested within, such that many rural community profiles are characterized by poverty and bilingualism (21). These characteristics are often perceived to lead to adverse effects, explicitly in migrant boomtowns (i.e., increased crime rates, greater need for social services, and so-called native “white flight”); however, the literature does not confirm these perceptions (19, 21). In sum, the sociodemographic characteristics of rural communities in the Great Plains suggest an oversimplified typology of communities where some rural communities are concentrated White, and some are concentrated Hispanic/Latino, often influenced by the community’s industry and employment structures.

The focus here is to examine the PA of children who are members of understudied rural “migrant boomtown” communities with a concentration of Hispanic/Latino children. Barker’s work on rural communities defined communities as systems of behavior settings, in which ecological units bounded by time and space drive patterns of behavior within a replicated social structure (22). Recently, this setting approach has been described as the community “wellness landscape” of settings that drives population PA outcomes (23). Rural behavior settings are places that provide a heterogeneous array of child adult-led services, such as afterschool programs, youth clubs, and sports (15, 24).

One community setting frequented by children, comprised of games and practices, is the sport setting. The production of the community landscape of youth sport settings within the U.S. has long institutional history (25). As an institution, organized youth sport (YS) has evolved from low-cost, positive youth development practices teaching so-called “American” values of cooperation, self-control, and respect for authority, to, at times, highly competitive, performance-based operations within for-profit entities supporting a win-at-all-costs mentality (26, 27). Despite this shift, YS, defined by the American Academy of Pediatrics as “PA that is directed by adult or youth leaders and involves rules and formal practice and competition” (28), continues to be a popular avenue to accumulate PA and other health benefits (29–31). Increasing sport participation among children and adolescents is also included as a *Healthy People 2030* PA objective (4), although, the current YS system’s pay-to-play model has erected barriers to participation, disproportionately impacting children from minority groups (32, 33). For example, Kellstedt and colleagues found family income was a significant (*p* < 0.05) predictor of YS participation among predominantly White children (*n* = 235) living in rural communities, in which children with full-pay lunch status were almost four times more likely to participate than peers with free or reduced lunch (OR = 3.91, 95% CI = 1.95–7.8) (34). Additionally, the authors found YS participants accumulated significantly more daily average minutes of MVPA than non-participants (84.9 min vs. 78.1 min, *p* < 0.05), which is consistent with the literature (35–37). However, further investigation of the impact of race, ethnicity, and the combination of these characteristics (i.e., ethnoracial status) on YS participation and MVPA within increasingly diverse rural American communities has been recommended, as cost may not be the only barrier for entry (e.g., lack of programming, facilities, and transportation limitations) into these and other social systems of interaction (11, 34).

It is well established that childhood PA is vital, valuable, and lacking, and the places where children live, learn, play, and age disproportionately impact this essential, modifiable health behavior. There is an opportunity to examine the influence of children getting more PA through YS participation (38); yet, there is a gap in understanding the sociodemographic characteristics related to YS participation in under-researched and under-resourced ethnoracially diverse rural communities and how these factors interact to influence PA outcomes (34, 39). The goals of this study were to (1) examine the association between participation in adult-led organized YS opportunities by grade, sex, ethnoracial status, and family income, and (2) determine the influence of these factors (grade, sex, ethnoracial status, and family income) and organized YS participation on PA levels of children living in ethnoracially diverse rural communities.

## Methods

### Study design

This study was a cross-sectional sub-study of the Wellscapes Project. Wellscapes is a two-wave staggered-start community randomized trial (ClinicalTrials.gov Identifier: NCT03380143, 20/12/2017) and social epidemiology study of four rural U.S. Midwestern communities aimed at establishing a multilevel system infrastructure to increase youth population PA (40). The protocol for all study activities was approved by the Institutional Review Board at the University of Nebraska Medical Center (IRB #446-18-EP, IRB #439-18-EX).

In Wave 1 (fall 2018–spring 2020), two rural, predominantly non-Hispanic White communities were recruited. In Wave 2 (fall 2021–spring 2023), two rural communities with a majority of Hispanic children were targeted for recruitment based on community proximity to urban areas (i.e., distance greater than 10 miles from an urbanized area, classified as rural distant and rural remote) (41), school districts with a concentration (≥50%) of Hispanic children enrolled in 3rd through 6th grades (i.e., approximately 8 to 12 years of age) (42), and population size (i.e., less than 3,500 residents). Additional inclusion criteria for Wave 2 communities included the following: had one public high school, offered multiple adult-led group opportunities (e.g., youth club meetings, YS team practices, etc.) for children in 3rd through 6th grades, independent completion of a community health needs assessment with prioritization of obesity prevention in a community health improvement plan, and agreement by the local health department and public school district to participate in the study.

### Study sample

The present study reports on spring 2022 population PA and corresponding contextual data collected during the Wellscapes Wave 2 baseline year (fall 2021–spring 2022). With permission from the school districts (*n* = 4; 2 public and 2 private), administration, and staff, all 3rd through 6th-grade public education classrooms (*n* = 16) and private education classrooms (*n* = 4) across the communities (*n* = 2) were eligible to participate in Wave 2’s social epidemiology study. This consisted of the administration of an online PA surveillance instrument, available in English and Spanish, near the beginning and end of each school year during designated class time. The instrument is comprised of the validated Youth Activity Profile (YAP) (43), an online assessment explicitly designed for school-based evaluations of youth PA with 15 items that children and adolescents can complete in 15–20 min (44, 45). Students in 4th grade and up can complete the YAP independently (46, 47); however, children as young as 3rd grade can complete the YAP with adult supervision as documented in the National Cancer Institute’s Family Life, Activity, Sun, Health, and Eating (FLASHE) Study (48). The instrument also includes supplemental out-of-school organized activity participation questions from the National Survey of Children’s Health 2017–2018 (NSCH) (49). We specifically concentrated on the second administration of the instrument in spring 2022 to capture children’s PA behaviors that occurred during the school year and maintain consistency with work from Wave 1. Previous publications have documented the protocol (50) and summarized descriptive PA and YS participation patterns at the population level in the Wave 1 communities (34).

During spring 2022, 97% of 3rd through 6th-grade children across both communities (*n* = 281) participated in the social epidemiology study by completing the PA surveillance instrument. This study primarily focused on a subset of those children (*n* = 215) who had informed parental and guardian consent. This consent, distributed in English and Spanish, was obtained to pair children’s school sociodemographic data (e.g., date of birth, sex, race, ethnicity, and free and reduced lunch status) with their respective PA data, including surveillance instrument responses, based on a Data Sharing Agreement (DSA) established with participating schools (50).

### Study procedures

The research team trained school administrators and teachers to administer the online instrument as an in-class educational experience (e.g., to learn about PA and sedentary behaviors) and provided ongoing technical assistance. The instrument was shared via a secure, web-based application designed to support data capture for research studies hosted at the University of Nebraska Medical Center, REDCap (Research Electronic Data Capture) (51, 52). Coinciding with the conclusion of the Wave 2 baseline year, the instrument was administered during the end of April through early May 2022 (43, 53). Children were guided through the online PA surveillance instrument with support from teachers and school staff and self-reported grade, sex, organized activity participation, and PA and sedentary behaviors. All 3rd through 6th-graders completed the YAP and supplemental questions within a classroom setting using the school’s media resources.

### Measures

#### Sociodemographic characteristics

Individual sociodemographic characteristics in this study included grade, sex, ethnoracial status, and a proxy for family income (i.e., lunch status). When completing the YAP, children were asked to self-report grade (i.e., 3rd, 4th, 5th, or 6th grade) and sex (i.e., male, female, not listed, or prefer not to answer). The DSA permitted the school to provide additional sociodemographic information obtained from school enrollment records, including student name, identification number, date of birth, grade, sex, race, ethnicity, and family free and reduced-price lunch status (FRLS). The school shared these data on a secure platform with the research team, and members of the research team paired de-identified school enrollment data with YAP data for the subsample of children whose parents and guardians consented to the community trial (50). Variables of interest were based on enrollment data and included grade (i.e., 3rd, 4th, 5th, or 6th grade) and sex (i.e., male or female).

Ethnoracial status was created by combining school recorded ethnicity (i.e., non-Hispanic or Hispanic) and race (i.e., White, Black/African American, American Indian/Alaska Native, Asian, Multiple Races/Mixed, or Unknown/did not report). This variable was further dichotomized to include two groupings: (1) non-Hispanic White and (2) Hispanic and racially diverse (including all children identified as Hispanic regardless of race, and children identified as non-Hispanic and any race other than White), for data quality based on small sample sizes among race-related classifications (54). A family income variable was dichotomized based on FRLS. Consistent with our previous work, children with FRLS were considered “lower income,” and children with full pay lunch status were considered “higher income” (34, 55). Within the constraints of the student enrollment records and DSA, we acknowledge lunch status is a sufficient proxy for family income based on the available data (56).

#### Youth sport participation

Participation in YS was determined using the supplemental NSCH questions added to the YAP (49). Specifically, these items assessed past-year participation in out-of-school activities, including daily afterschool programs, sports teams or sports lessons, clubs or organizations, and other organized activities or lessons (e.g., music, dance, language, or other arts). The present analyses relied on child self-reported “yes” or “no” responses to the following question: “During the past 12 months, did you participate in a sports team or take sports lessons after school or on weekends?” (49). If a child answered “yes,” they were included as a YS participant.

#### Moderate-to-vigorous physical activity

The primary outcome variable of children’s MVPA was estimated using published calibration equations developed specifically for the online version of the YAP (43). In the YAP, children respond to five questions about in-school PA and five questions related to out-of-school PA. The published algorithms were applied to the 10 PA items to create estimates of in-school PA, out-of-school PA, and weekend PA. The YAP items used to estimate weekday (Monday – Friday) in-school MVPA asked children to report how many days (0, 1, 2, 3, or 4 to 5 days) they walked or biked to school and how many days they walked or biked from school, how often they were running and moving as part of the planned games or activities during physical education, and how often they were playing sports, walking, running, or playing active games during recess (with response options including 0 = no physical education or recess, 1 = almost none of the time, 2 = a little bit, 3 = a moderate amount, 4 = a lot, or 5 = almost all of the time) (43). The YAP items used to estimate weekday out-of-school MVPA asked children to report how many days (0, 1, 2, 3, or 4 to 5 days) they did some form of PA for at least 10 min (1) before school (between 6:00–8:00 a.m.), (2) after school (3:00–6:00 p.m.), and (3) in the evening (6:00–10:00 p.m.), Monday – Friday (43). Weekend MVPA was estimated using the YAP items that asked about amounts of PA (no activity [0 min], small amount of activity [1 to 30 min], small-to-moderate amount of activity [31 to 60 min], moderate-to-large amount of activity [1 to 2 h], and large amount of activity [more than 2 h]) accumulated on Saturday and Sunday (e.g., exercise, work/chores, and family outings) (43). The average daily PA level was computed using a weighted average of weekday (both in-school and out-of-school) PA (5 days) and weekend PA (2 days). Past work with the YAP has shown that in-school and out-of-school PA values from the YAP were within 23 and 21% of values derived from an objective PA monitor, respectively, based on mean absolute percentage error calculations (43).

### Data analyses

Descriptive statistics were used to summarize children’s participation in YS for the primary social epidemiology study (*n* = 281) and the community trial sub-study (*n* = 215). Mixed-models were used to analyze the dichotomous outcome (i.e., 1 = Yes; 0 = No) of YS participation, and the continuous outcome of average minutes of daily MVPA. For all models, community was included as a random effect (34). SAS/STAT software, version 9.4 (^©^ 2002–2012) of the SAS System for Windows (Cary, NC), was used for all analyses. PROC GLIMMIX was performed to examine the dichotomous outcome of YS participation with the fixed effects of grade, sex, ethnoracial status, and family income. Interactions between the effects of grade, sex, ethnoracial status, and family income on YS participation were also explored. PROC MIXED was used to examine average minutes of daily MVPA with the fixed effects of grade, sex, ethnoracial status, family income, and YS participation. Similarly, interaction effects of grade, sex, ethnoracial status, family income, and YS participation on average time in MVPA were examined. Backward elimination of non-significant interactions based on statistical significance of *p* < 0.05 was used where non-significant higher order interactions were eliminated first, and then the models were refit (57, 58).

## Results

Descriptive characteristics of children in the Wellscapes social epidemiology study and those who participated in the Wellscapes community trial are found in Table 1. A total of 281 3rd through 6th-grade children (97% of total student population) completed the YAP and supplemental NSCH questions, and of those, 215 children had parental and guardian consent to be included in the community trial. Among the community trial participants, more children identified as Hispanic and racially diverse than non-Hispanic White, had lower family income than higher family income, and participated in YS opportunities than did not.

**Table 1:** Descriptive characteristics of children.

	Social epidemiology study participation	Community trial participation
	*n* (%)	*n* (%)
Child Participants	281	215
*Grade*		
3rd	74 (26.3)	58 (27.0)
4th	64 (22.8)	52 (24.2)
5th	75 (26.7)	50 (23.3)
6th	68 (24.2)	55 (25.6)
*Sex*		
Male	131 (46.6)	93 (43.3)
Female	150 (53.4)	122 (56.7)
*Ethnoracial Status*		
Hispanic and Racially Diverse*	–	133 (61.9)
Non-Hispanic White	–	82 (38.1)
*Family Income*		
Lower (Free/Reduced)	–	112 (52.1)
Higher (Full Pay)	–	103 (47.9)
*Sport Participation in Past 12 Months*		
Yes	137 (48.8)	117 (54.4)
No	144 (51.3)	98 (45.6)
*Includes the following:		
Hispanic, White	–	51 (23.7)
Hispanic, Black/AA, AIAN, Asian, or Multi-Race	–	26 (12.1)
Hispanic, Race Reported as Unknown/Did Not Report	–	44 (20.5)
Non-Hispanic Black/AA, AIAN, Asian, or Multi-Race	–	12 (5.6)

Black/African American (Black/AA); American Indian/Alaska Native (AIAN).

### Youth sport participation

**Figure 1:**
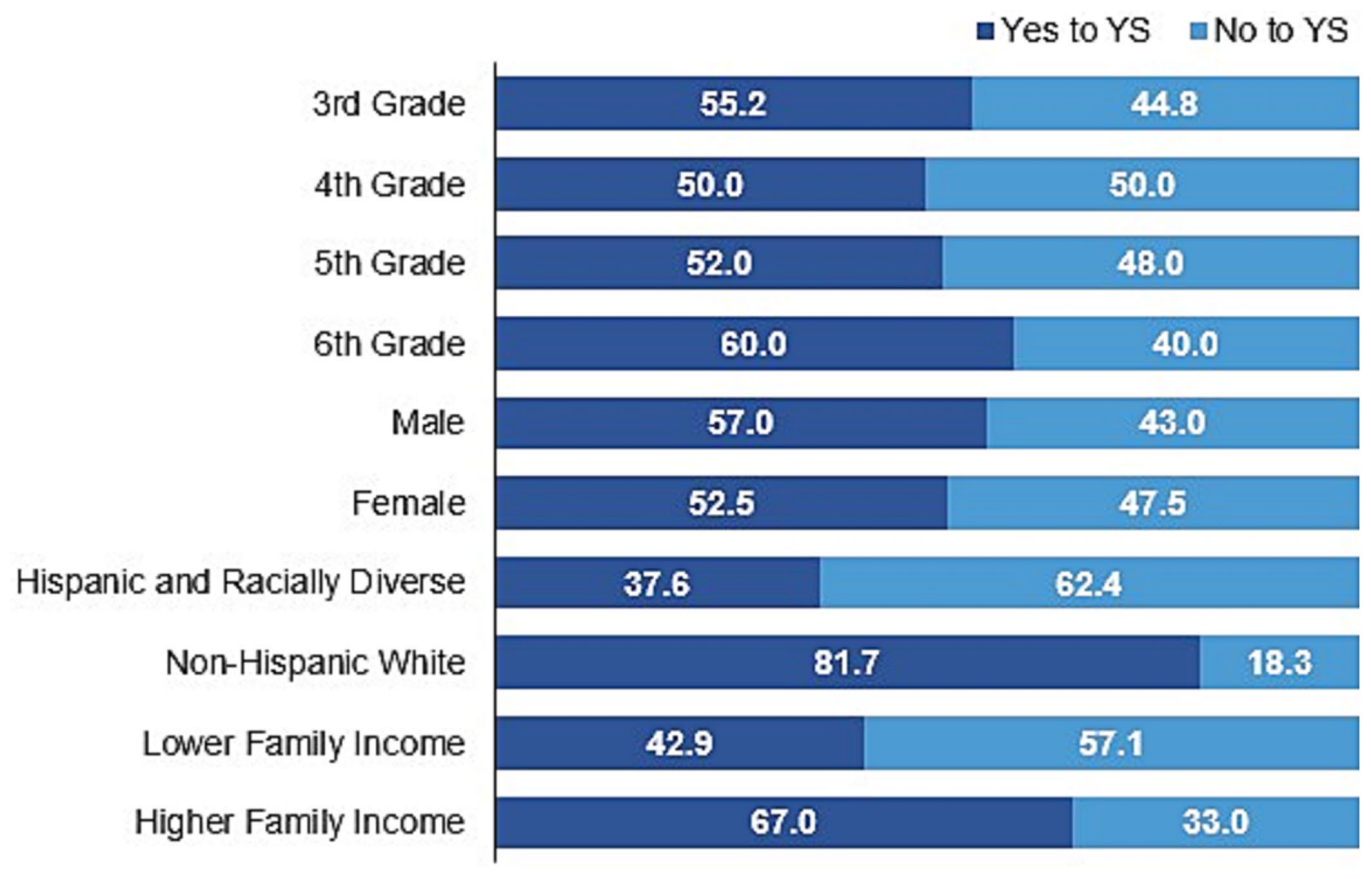
Percentage of children from community trial that participated in youth sports (YS) in the past 12 months.

Figure 1 highlights the proportion of children in the community trial reporting YS participation based on sociodemographic characteristics, and Table 2 shows the results from the mixed-model regression predicting YS participation among trial participants. Non-Hispanic White children were over five times more likely to participate in YS than Hispanic and racially diverse peers (OR = 5.54, 95% CI = 2.64–11.61, *p* < 0.001). Additionally, children with higher family income were almost twice as likely to participate in YS than children with lower family income (OR = 1.94, 95% CI = 0.98–3.86), however, this finding was not significant (*p* = 0.058). There were also no significant differences in the likelihood of participating in YS by grade level or sex, and significant interactions were not found.

**Table 2:** Likelihood of participating in youth sport opportunities by characteristics of children.

Characteristics of children	Odds ratio	95% CI	*p*-value
*Grade*			
3rd	Reference (1.00)			
4th	0.74	(0.31–1.77)	0.501
5th	0.60	(0.24–1.51)	0.277
6th	1.34	(0.56–3.23)	0.511
*Sex*			
Male	Reference (1.00)		
Female	0.88	(0.46–1.68)	0.707
*Ethnoracial Status*			
Hispanic and Racially Diverse	Reference (1.00)		
Non-Hispanic White	5.54*	(2.64–11.61)	<0.001
*Family Income*			
Lower (Free/Reduced)	Reference (1.00)		
Higher (Full Pay)	1.94	(0.98–3.86)	0.058

CI, confidence interval.

* Odds ratio is significant (*p* < 0.05).

### Moderate-to-vigorous physical activity

Table 3 presents least squares means and significant differences in daily MVPA by fixed effects of grade, sex, ethnoracial status, family income, and YS participation. There were significant main effects on daily MVPA for grade (*F*(3, 203) = 24.03, *p* < 0.001), sex (*F*(1, 203) = 195.46, *p* < 0.001), ethnoracial status (*F*(1, 204) = 6.23, *p* = 0.013), and YS participation (*F*(1, 201) = 13.47, *p* < 0.001). Compared with 3rd, 4th, and 5th graders, 6th graders reported significantly less time in daily MVPA. Additionally, males reported significantly more daily minutes of MVPA (mean = 86.6 ± 3.1 min/day) compared with females (mean = 60.0 ± 3.0 min/day). Children identified as non-Hispanic White also reported significantly more minutes of daily MVPA than peers identified as Hispanic and racially diverse (mean = 76.2 ± 3.2 min/day and 70.4 ± 3.0 min/day, respectively). Significant interaction effects on daily MVPA between grade and ethnoracial status (*F*(3, 204) = 3.04, *p* = 0.030) were also found, as highlighted in Table 4. Fourth and 6th graders identified as non-Hispanic White reported significantly more minutes of daily MVPA than their respective grade-level matched Hispanic and racially diverse peers, with an approximate mean difference of 9.8 min and 12.3 min, respectively. Conversely, 5th graders identified as Hispanic and racially diverse reported significantly more minutes of daily MVPA (mean = 75.1 ± 3.9 min/day) than 6th graders identified as non-Hispanic White (mean = 66.0 ± 4.2 min/day). In addition, Hispanic and racially diverse 3rd graders reported more minutes of daily MVPA compared with non-Hispanic White 3rd graders (mean = 83.0 ± 3.6 min/day and 80.4 ± 4.2 min/day, respectively), although the difference was not significant.

**Table 3:** Least squares means estimates of average total minutes of daily MVPA by characteristics of children.

Characteristics of children	MVPA, Adjusted Mean (95% CI)	Differences (*p* < 0.05)
*Grade*		Differences^a^ (*p* < 0.05)
a. 3rd	81.7 (64.5–99.0)	b, d
b. 4th	74.8 (58.5–91.1)	a, d
c. 5th	76.8 (59.7–93.8)	d
d. 6th	59.8 (43.1–76.5)	a, b, c
*Sex*		Differences^b^ (*p* < 0.05)
a. Male	86.6 (61.7–111.4)	b
b. Female	60.0 (30.9–89.1)	a
*Ethnoracial Status*		Differences^c^ (*p* < 0.05)
a. Hispanic and Racially Diverse	70.4 (41.5–99.3)	b
b. Non-Hispanic White	76.2 (56.2–96.1)	a
*Family Income*		Differences^d^ (*p* < 0.05)
a. Lower (Free/Reduced)	72.9 (48.1–97.7)	–
b. Higher (Full Pay)	73.6 (48.0–99.3)	–
*Youth Sport Participation*		Differences^e^ (*p* < 0.05)
a. Yes	77.4 (47.9–106.9)	b
b. No	69.1 (48.1–90.2)	a

MVPA, moderate-to-vigorous physical activity; CI, confidence interval.

a Significance from mixed effects model (e.g., “a” denotes difference from 3rd Grade).

b Significance from mixed effects model (e.g., “a” denotes difference from Male).

c Significance from mixed effects model (e.g., “a” denotes difference from Hispanic and Racially Diverse).

d Significance from mixed effects model (no significant difference found).

e Significance from mixed effects model (e.g., “a” denotes difference from Yes to Youth Sport Participation).

**Table 4:** Least squares means estimates of average total minutes of daily MVPA by grade interactions with ethnoracial status.

Characteristics of children	MVPA, adjusted mean (95% CI)	Differences^a1^ (*p* < 0.05)
*Non-Hispanic White*		
a. 3rd grade	80.4 (69.0–91.9)	d, f, h
b. 4th grade	79.7 (68.3–91.0)	d, f, h
c. 5th grade	78.5 (66.5–90.5)	d, f, h
d. 6th grade	66.0 (54.6–77.3)	a, b, c, e, g, h
*Hispanic and Racially Diverse*		
e. 3rd grade	83.0 (68.9–97.2)	d, f, g, h
f. 4th grade	69.9 (56.5–83.3)	a, b, c, e, h
g. 5th grade	75.1 (62.8–87.3)	d, e, h
h. 6th grade	53.6 (39.9–67.3)	a, b, c, d, e, f, g

MVPA, moderate-to-vigorous physical activity; CI, confidence interval.

a1 Significance from mixed effects model (e.g., “a” denotes difference from Non-Hispanic White in 3rd grade).

**Figure 2:**
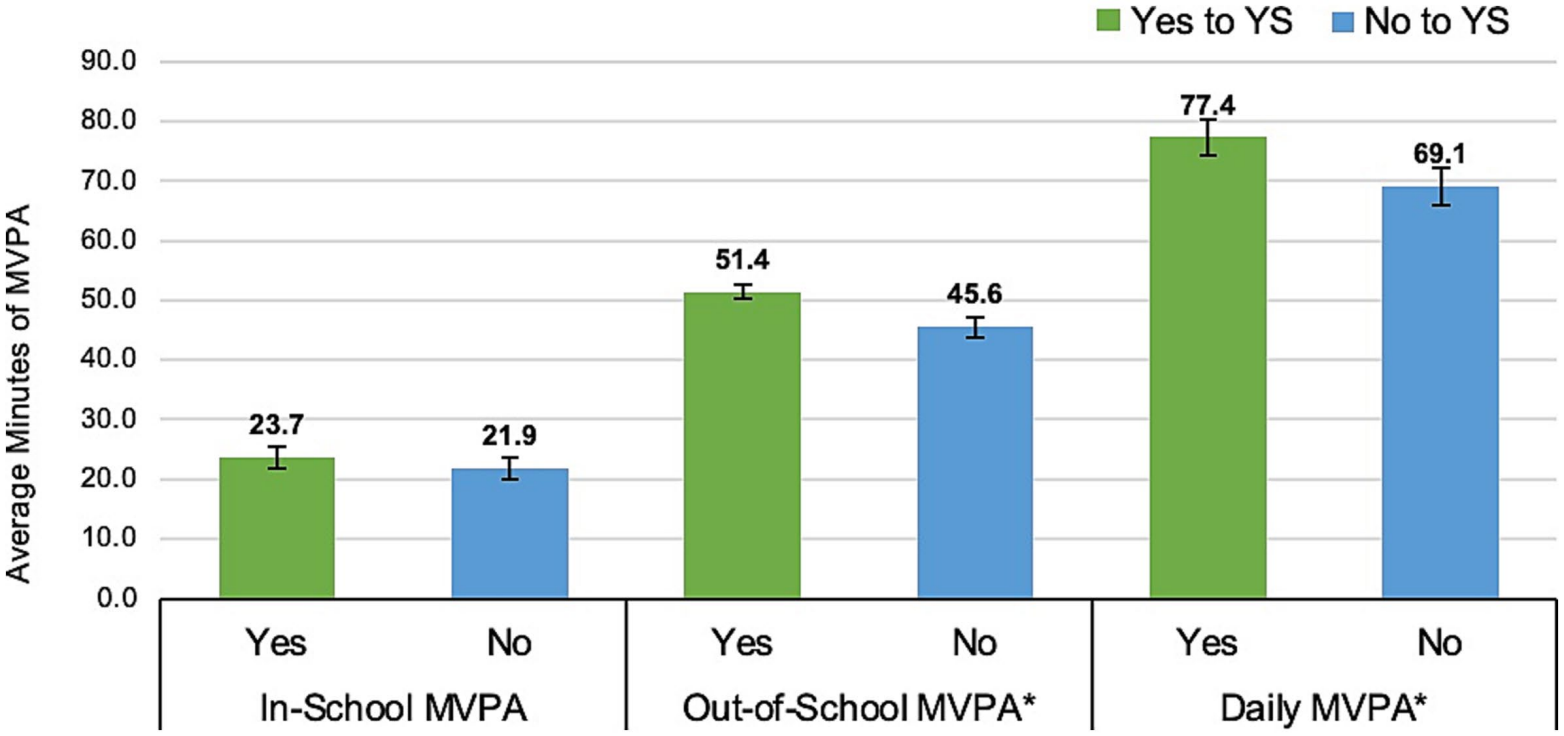
Differences in average minutes of MVPA based on youth sport (YS) participation. *Note*. Error bars represent standard error (*p < 0.05). In-and-Out-of-School MVPA includes average daily minutes of MVPA per weekday during school time and out-of-school time, and Daily MVPA includes average daily minutes of MVPA per weekday and weekend days.

There were no significant main effects on daily MVPA by family income (*F*(1, 204) = 0.13, *p* = 0.723). Children participating in YS opportunities reported significantly more average total minutes of daily MVPA (mean = 77.4 ± 3.0 min/day) than those not participating (mean = 69.1 ± 3.2 min/day). Figure 2 shows differences between YS participation and average minutes of MVPA, including differences in total daily minutes of MVPA per weekday and weekend days (Monday–Sunday), and differences in minutes of MVPA during in-school and out-of-school time per weekday (Monday–Friday). Children who participated in YS opportunities had similar in-school MVPA than children who did not participate in YS (23.7 min and 21.9 min, respectively, *p* = 0.061). However, YS participants reported significantly (*p* = 0.006) more daily minutes of out-of-school MVPA per weekday (mean = 51.4 ± 1.2 min/day) than those who did not participate (mean = 45.6 ± 1.7 min/day).

## Discussion

The current study examined the association of grade, sex, ethnoracial status, and family income on YS participation and their impact on the average daily MVPA of children living in ethnoracially diverse rural communities. In this study, YS participation included involvement in a sports team or sports lessons after school or on weekends. A key finding was that approximately 54% of children reported participating in organized YS opportunities within the past 12 months. Although this percentage is consistent with nationally representative data for children and adolescents aged 6–17 years from 2020 (59), which the authors asserted may not be reflective of changes in participation based on the COVID-19 pandemic, it is lower than the 80% of rural children engaged in YS as reported by Kellstedt et al. (34) based on data collected in spring of 2019 (34). This difference may be a difference among communities or, as the literature suggests, YS participation may still be rebounding to pre-pandemic levels, while recognizing significant participation disparities based on sociodemographic characteristics (e.g., sex and race) existed during the pandemic (60, 61). For example, Fleming et al. (61) found that ethnoracially diverse (i.e., Asian, Latinx, and Multiracial) and rural children spent significantly less time in YS settings during the pandemic than comparative peers (i.e., White and urban children, respectively), and as a result, may be experiencing a “double jeopardy” effect in returning to organized sport opportunities given participation disparities that existed prior to the pandemic (61). Similarly, organized YS opportunities themselves have also been impacted by the COVID-19 pandemic (62, 63), and in rural communities, where the availability and access to organized YS have been shown to be more limited pre-pandemic (11, 64), previously existing opportunities may have struggled to survive; in turn, widening participation gaps for already vulnerable populations. Despite these gaps and the need for more empirical research examining the impact of community disruptions (e.g., COVID-19) on organized YS systems, many (>50%) children post-system disruption in this study reported YS participation. Thus, rural community improvement of the local YS system has the potential to impact population health outcomes, including childhood PA, provided the proportion of children accessing YS opportunities (34, 35).

Characterizing children by grade level and sex revealed no significant differences in organized YS participation, which is consistent with 2020 findings reported in a recent National Center for Health Statistics’ Data Brief (59). Specifically, Black and colleagues found that while the percentage of children (aged 6–17 years) that participated in sports was higher for males compared with females (56.1 and 52.0%, respectively) and for children aged 12–17 years compared with children aged 6–11 years (55.6 and 52.5%, respectively), the observed differences were not significant (59). Similarly, in this study, males and 6th graders (typically aged 11–12 years) from the community trial had higher rates of YS participation (57.0 and 60.0%, respectively) than females (52.5%) and 3rd (55.2%), 4th (50.0%), and 5th (52.0%) graders, collectively, but the results were non-significant. These findings have also been observed in other studies (65), including those exploring midwestern rural areas (34, 66), which may be highlighting dramatic shifts in rural community cultural expectations for and acceptance of female YS participation (e.g., reversing sex and gender-based inequalities in organized sport structures), or, more practically, the need to solve local issues of supply and demand regardless of a child’s age or sex, or any embedded structural inequities (e.g., need to fill team rosters with limited supply of participants) (67–69). Future research should continue monitoring YS participation patterns based on grade and sex, while being mindful to explore organized YS participation patterns in children beyond 6th grade, where middle- and high-school sanctioned sports impact the community wellness landscape and sport dropout percentages often increase (70). Additionally, calls to include the examination of gender and sexual identity and sport warrant further investigation, particularly with the rise in gender and sexual fluidity among children and adolescents (71, 72). Advocates recommend policymakers develop more inclusive, evidence-based sport policies (e.g., Canadian Centre for Ethics in Sport), to create safe sport environments for all athletes, regardless of identity (72). Although, we acknowledge the difficulty of this responsibility in light of current laws restricting gender-affirming care and recent bans on transgender sport participation (73).

Our work also revealed that rural children classified by family income were not significantly different in organized YS participation, but ethnoracial status was significantly associated with participation. Specifically, children identified as non-Hispanic White were over five times more likely to participate in YS than Hispanic and racially diverse peers (OR = 5.54, 95% CI = 2.64–11.61, *p* < 0.001). This finding is noteworthy since much of the literature supports disparities in YS participation based on family socioeconomic status (33, 34, 65, 74), whereas studies primarily comparing ethnic backgrounds (e.g., Hispanic and non-Hispanic) of children with YS participation are scarce. Racial and ethnic sport disparities have been documented among secondary school children (i.e., grades 8th, 10th, and 12th) (65) and children aged 6–17 years (59) living in the U.S., as well as among children living in other countries (75–77). A cross-sectional study by Wijtzes et al. (75), examining social inequalities in young children’s (*n* = 4,726; average age of 6 years) sports participation and outdoor play in the Netherlands, found that all ethnic minority children (i.e., Surinamese-Creole, Surinamese-Hindustani, Dutch Antillean, Cape Verdean, Turkish, and Moroccan) were more likely to not participate in sports compared with native ethnic majority Dutch children, with the highest odds for Turkish children (OR = 3.16, 95% CI = 2.51–3.98, *p* < 0.05). However, when adjusting for family social economic position, many ethnic-related associations became non-significant (75). This attenuation, despite glaring differences in study characteristics, may have occurred in the present study if a more comprehensive indicator of social economic status was used in place of FRLS, but we must not downplay the clear association found between ethnoracial status and YS participation. We must also continue studying the social and structural factors underlying this inequality (e.g., cultural norms, discriminatory processes, and acculturation characteristics) (77), especially in rural communities with growing diversity, to understand if the differences in YS participation based on ethnoracial status stem from broader inequities (e.g., lack of culturally-relevant sport opportunities, parental time opportunities, unnecessary documentation for registration, and inadequate communication translations), possibly leading to “unfair and avoidable differences” in health outcomes (69). Thus, examining the root causes of sport and PA-related inequities is vital to advance health equity, including support for more equitable organized YS opportunities where all children are encouraged to participate.

There are many benefits associated with persistent YS participation (78, 79), and although this study did not examine the breadth nor continuation of children’s organized sport participation in subsequent years, it did confirm YS plays a significant role in accumulating daily PA. Namely, we found that YS participants had significantly (*p* < 0.01) greater MVPA than non-participants, by approximately 8 min more per day and 6 min more per weekday out-of-school time (mean_diff_ = 8.3 ± 2.3 min/day; mean_diff_ = 5.8 ± 2.1 min/day, respectively), which are not small amounts when accumulated over the span of a week. Yet, differences in in-school MVPA between YS participants and non-participants were non-significant (mean_diff_ = 1.8 ± 0.9 min/day), suggesting organized YS opportunities provide a meaningful contribution to PA, particularly for rural children outside of the school day. These findings are supported in the literature (34, 35, 80), but warrant further investigation, as type and level of sport opportunities (e.g., recreational and competitive soccer, volleyball, and basketball), context and location of practice sessions (e.g., outdoor full-field scrimmage or indoor walkthrough the day before a game), and leader-level characteristics and routines (e.g., volunteer or paid coaches, following predefined practice sessions developed by organizational authority or having flexibility to change sessions based on participant feedback), are highly variable, especially in under-resourced rural communities, and influence the amount of time children spend being active (81, 82). Thus, future research needs to assess the explicit and implicit rules of specific sport organizing bodies, among other factors, to determine how different sport practices may be promoting or discouraging associated outcomes like PA.

Another key finding in our study confirmed what is already known about the patterns of childhood PA characterized by grade and sex (83–86). Specifically, we found significant differences in daily MVPA by grade and sex. Compared with 3rd, 4th, and 5th graders, 6th graders reported the lowest average daily minutes of MVPA, with the largest mean difference between 3rd and 6th graders (i.e., approximately 22 min/day). In addition, males reported higher amounts of daily MVPA than females (mean_diff_ = 26.6 ± 1.9 min/day). Lower levels of MVPA as children age and lower levels of female MVPA have been presented in other studies, but reasons for these differences have been mixed (87, 88). Sport participation has been cited as a factor contributing to PA disparities (89), but our study does not show a significant decline in YS participation by grade nor a significant difference between male and female participation rates. Therefore, similar to the results shared by Kellstedt et al. (34), YS does not appear to be a driver of PA differences among rural children with these sociodemographic characteristics and begs a broader examination of other environments children frequent (e.g., home, school, youth clubs, etc.) to uncover why consistent PA disparities by grade and sex exist. Within the community wellness landscape of settings, the structure of the school day, for instance, may be driving the grade-related drop in PA, as older children’s access to recess and physical education classes are often limited or nonexistent compared to opportunities for younger children (65, 90). Thus, educators (e.g., school administrators and teachers) can play a critical role in improving child population health by finding ways to insert time devoted to PA (i.e., PA sessions) during the school day. Additionally, Stoepker and Dzewaltowski (91), although interested in out-of-school time leaders, provide elements of a novel data coaching intervention that may assist teachers in this PA-promotion practice. As a result, investigating the ecological processes and patterns that influence PA behaviors in non-sport rural settings is fundamental to reducing PA-related health disparities.

Organized YS, however, may be a driver of the significant difference in daily MVPA observed by ethnoracial status (*p* = 0.013). We acknowledge the approximate 6-min difference found between non-Hispanic White and Hispanic and racially diverse children should be interpreted with caution provided the reliance on self-reported PA. However, our results are consistent with earlier evidence for racial and ethnic PA disparities (59, 86, 92), even if estimates suggest children are meeting the daily PA guidelines. A 2021 mixed methods study exploring PA patterns and parental influences on rural Latino school-aged children (*n* = 27 3rd–5th graders) also found 100% of children met PA guidelines as measured by accelerometry (93), but parents (*n* = 31) were quick to acknowledge PA barriers and challenges for children. Specifically, the authors highlighted homework, technology (e.g., computer games and television), and a lack of engagement in organized team and league sports after school as major barriers to being active. Aside from children not being interested in playing sports, parents recognized cost and transportation as additional YS barriers but also shared their children engaged in more unstructured activity outside during afterschool hours (93). As a result, future whole-of-community interventions (6) aimed at increasing daily PA for ethnoracially diverse children living in rural communities may need to both advocate for low-cost and easily accessible PA opportunities, like YS programs, and also encourage more unstructured free play engaging the entire family.

Unlike grade, sex, and ethnoracial status, family income did not significantly impact children’s average daily MVPA. It is often assumed children living in poverty are more likely to be less active than children from higher-income families, but evidence does not consistently reflect this pattern (83, 94). This inconsistency has also been noted for children living in rural communities (34, 95). Like rural children identified as Hispanic and racially diverse, children from low-income families may be more active while outdoors in unstructured free play, thus, offsetting the PA accumulated in more structured activities among higher-income peers (95, 96). Additionally, in this study, family income did not have a significant effect on YS participation, so sport opportunities may be more accessible regardless of cost, or other factors, such as issues of supply and demand and a desire for community belonging, may encourage YS participation despite cost (67, 97). However, more research is warranted since family income can rapidly change, thus, confirming the need for a more comprehensive indicator of social economic status, like social class (98, 99), to determine if PA disparities exist. This is particularly relevant in rural communities with growing ethnoracial diversity where members of certain classes often have more power and control over others based on factors like wealth, education, and occupation.

We also observed significant interactions between grade and ethnoracial status for the primary outcome of children’s average minutes per day of MVPA. In most cases, children identified as non-Hispanic White reported more minutes of daily MVPA than Hispanic and racially diverse peers; however, differences were only significant for the 4th and 6th grade comparison groups. Conversely, Hispanic and racially diverse 3rd graders reported more minutes of daily MVPA compared with non-Hispanic White 3rd graders, although the difference was not significant. A study by Barr-Anderson et al. (100) supports a race/ethnicity by time interaction; however, the authors found no significant PA differences between Hispanic youth and White and Black youth at 5th, 6th, and 7th grade timepoints but acknowledge the sample size for Hispanic youth (*n* = 72) was significantly smaller than the other racial subgroups (Black, *n* = 275; White, *n* = 296). Despite this limitation, the study also reported ethnoracial differences in the factors that influenced total PA and age-related changes in PA. For example, among Hispanic youth, parent report of seeing other children outdoors was positively associated with total PA, while parent-reported child sport participation was one factor positively associated with total PA among White children (100). This, according to Hasson (101), “sheds light on the complexity of physical activity behavior and demonstrates that one size does not fit all when attempting to address physical inactivity in a racially and ethnically diverse group of children and adolescents.” We would add these factors might also help to shine light on why non-Hispanic White children were over five times more likely to participate in YS than Hispanic and racially diverse peers, but additional exploration is necessary given the gaps in the literature (100).

### Limitations

Provided the diversity of organized YS opportunities within local communities and across the U.S. and the fact our sample was limited to two rural Great Plains communities and their respective YS systems, the results of this study should be interpreted in light of its limitations. The children in the communities from this wave of the Wellscapes Project were primarily Hispanic and racially diverse and may not be representative of all rural American communities. However, results from the first wave are available and represent sociodemographic variability from Wave 2 (i.e., predominantly White rural communities) (34). Additionally, we did not account for cultural heterogeneity among Hispanic and racially diverse children and acknowledge the need to examine one’s country of origin, the amount of time in the U.S., and preferred language, explicitly as limiting materials (i.e., PA surveillance instrument and consent forms) to English and Spanish may have resulted in sampling and response biases (102). Next, there are limitations to the self-report measure of organized YS involvement and PA used in the present study. The YAP is designed to provide reasonably accurate group-level estimates of daily MVPA, but the conclusions are still based on youth self-reported data. An improved self-report instrument might also be more sensitive to the different types and number of sports played among YS participants, which may influence children’s PA and other quality of life factors (103). Future research should consider adopting a protocol similar to that defined by Essay and colleagues (23) to gain more objective data about children’s PA and participation in adult-led organized opportunities not limited to YS, as we have previously alluded to the importance of understanding the quality and quantity of organized opportunities and the PA opportunities nested within. However, obtaining an objective PA outcome measure (e.g., accelerometry) may result in a sample not fully representative of the community; plus, data collection efforts are resource intense (23).

Furthermore, we must acknowledge the limitation of the cross-sectional study design, in which a causal link between YS participation and PA cannot be established. Future experimental study designs should also consider rural children’s participation in non-sport out-of-school time organized activities, like afterschool programs, as well as time spent in unstructured activities (e.g., free play) to capture the larger community wellness landscape, and overall PA implications. In addition, our study’s definition of family income based on lunch status also warrants attention, as a more comprehensive indicator, like social economic status, may be more accurate (100). However, this classification is considered a sufficient proxy given the privacy requirements associated with school student enrollment records (56).

## Conclusion

Youth physical activity (PA) has declined over time, and opportunities for activity vary by where children live, learn, and play. Lack of PA among children living in rural communities is a documented public health problem, although much of the literature is constrained by the classic rural–urban dichotomy, failing to account for the complexity within communities. One such complexity is ethnoracial diversity, characteristic of Midwest migrant boomtowns. Another complexity is the heterogenous landscape of opportunities within communities, including organized sport settings. As a result, this study embraced this complexity and attempted to fill several significant gaps by examining sociodemographic factors related to youth sport (YS) participation and how these factors interact to influence PA among children living in ethnoracially diverse rural communities.

This study suggests that participating in organized YS plays a critical role in the daily MVPA of children living in ethnoracially diverse rural communities. However, disparities in YS participation and PA outcomes, primarily attributed to ethnoracial status rather than family income, assert the need for further investigation to determine who and what might be driving these phenomena. Additionally, our findings suggest the need to advocate for the removal of other, non-cost related YS participation barriers, such as policies and practices provoking exclusion (e.g., entry rules and gender stereotypes). Time spent in unstructured activities also warrants more research, as future findings may suggest these low-to-no cost opportunities may be more advantageous when considering population health outcomes across the lifespan. Furthermore, understanding the relationships between grade, sex, ethnoracial status, and family income on organized YS participation can also inform whole-of-community interventions targeting PA policy, systems, and environmental change strategies to help create more equitable and accessible behavior settings for children living in concentrated Hispanic rural communities. Ultimately, increasing YS participation, consistent with *Healthy People 2030* objectives, could make a significant impact on the PA and health of children living in ethnoracially diverse rural communities.

## Data availability statement

The raw data supporting the conclusions of this article will be made available by the authors, without undue reservation.

## Ethics statement

The studies involving humans were approved by the Institutional Review Board at the University of Nebraska Medical Center. The studies were conducted in accordance with the local legislation and institutional requirements. Written informed consent for participation in this study was provided by the participants’ legal guardians/next of kin.

## Author contributions

MJVS: Writing – original draft, Conceptualization, Methodology. AER: Writing – review & editing, Conceptualization, Formal analysis, Methodology. MAS: Writing – review & editing, Conceptualization, Methodology. DKK: Writing – review & editing, Conceptualization, Methodology. GJW: Writing – review & editing, Conceptualization, Methodology. RH: Writing – review & editing, Formal analysis. DAD: Writing – review & editing, Conceptualization, Formal analysis, Funding acquisition, Methodology, Supervision.

## References

[1] Janssen I Leblanc AG. Systematic review of the health benefits of physical activity and fitness in school-aged children and youth. Int J Behav Nutr Phys Act. (2010) 7:40. doi: 10.1186/1479-5868-7-40, PMID: 20459784 PMC2885312

[2] Bouchard C Blair SN Haskell WL. Physical activity and health. Human Kinetics. New York, NY: Routledge. (2018) 103–69.

[3] U.S. Department of Health and Human Services. Physical activity guidelines for Americans [internet]. 2nd ed. Washington, DC: U.S. Department of Health and Human Services (2018).

[4] Office of Disease Prevention and Health Promotion. Healthy people 2030. U.S. Department of Health and Human Services. (2020).

[5] Parsons T. The Social System (Major Languages). *2nd ed*. London: Routledge. (1991) 1–15.

[6] Essay AM Schlechter CR Mershon CA Fial AV Ellison J Rosenkranz RR. A scoping review of whole-of-community interventions on six modifiable cancer prevention risk factors in youth: a systems typology. Prev Med. (2021) 153:106769. doi: 10.1016/j.ypmed.2021.106769, PMID: 34416222

[7] Lee BA Sharp G. Ethnoracial diversity across the rural-urban continuum. Ann Am Acad Pol Soc Sci. (2017) 672:26–45. doi: 10.1177/0002716217708560, PMID: 31814626 PMC6897380

[8] Joens-Matre RR Welk GJ Calabro MA Russell DW Nicklay E Hensley LD. Rural-urban differences in physical activity, physical fitness, and overweight prevalence of children. J Rural Health. (2008) 24:49–54. doi: 10.1111/j.1748-0361.2008.00136.x, PMID: 18257870

[9] Moore JB Brinkley J Crawford TW Evenson KR Brownson RC. Association of the built environment with physical activity and adiposity in rural and urban youth. Prev Med. (2013) 56:145–8. doi: 10.1016/j.ypmed.2012.11.019, PMID: 23219761 PMC4761410

[10] Moore JB Beets MW Morris SF Kolbe MB. Comparison of objectively measured physical activity levels of rural, suburban, and urban youth. Am J Prev Med. (2014) 46:289–92. doi: 10.1016/j.amepre.2013.11.001, PMID: 24512868

[11] Yousefian A Ziller E Swartz J Hartley D. Active living for rural youth: addressing physical inactivity in rural communities. J Public Health Manag Pract. (2009) 15:223–31. doi: 10.1097/PHH.0b013e3181a11822 19363402

[12] McCormack LA Meendering J. Diet and physical activity in rural vs urban children and adolescents in the United States: a narrative review. J Acad Nutr Diet. (2016) 116:467–80. doi: 10.1016/j.jand.2015.10.024, PMID: 26685123

[13] Hall M Tach L Lee BA. Trajectories of Ethnoracial diversity in American communities, 1980-2010. Popul Dev Rev. (2016) 42:271–97. doi: 10.1111/j.1728-4457.2016.00125.x, PMID: 29398737 PMC5791753

[14] Bettenhausen JL Winterer CM Colvin JD. Health and poverty of rural children: an under-researched and under-resourced vulnerable population. Acad Pediatr. (2021) 21:S126–33. doi: 10.1016/j.acap.2021.08.001, PMID: 34740419

[15] Sandercock G Angus C Barton J. Physical activity levels of children living in different built environments. Prev Med. (2010) 50:193–8. doi: 10.1016/j.ypmed.2010.01.005 20083131

[16] Lichter DT. Immigration and the new racial diversity in rural America. Rural Sociol. (2012) 77:3–35. doi: 10.1111/j.1549-0831.2012.00070.x, PMID: 26478602 PMC4606139

[17] Lichter DT Johnson KM. A demographic lifeline? Immigration and hispanic population growth in rural America. Popul Res Policy Rev. (2020) 39:785–03. doi: 10.1007/s11113-020-09605-8

[18] Butler J Wildermuth GA Thiede BC Brown DL. Population change and income inequality in rural america. Popul Res Policy Rev. (2020) 39:889–11. doi: 10.1007/s11113-020-09606-7, PMID: 34744225 PMC8570540

[19] Brown DL Schafft KA. Rural people and communities in the 21st century: resilience and transformation. 2nd ed. Cambridge, UK: Polity (2018).

[20] Carr PJ Lichter DT Kefalas MJ. Can immigration save small-town America? Hispanic boomtowns and the uneasy path to renewal. Ann Am Acad Pol Soc Sci. (2012) 641:38–57. doi: 10.1177/0002716211433445

[21] Artz GM. Immigration and meatpacking in the Midwest. Choices. (2012) 27. Available at: http://www.jstor.org/stable/choices.27.2.10

[22] Barker RG. The stream of behavior: Explorations of its structure & content. East Norwalk: Appleton-Century-Crofts (1963).

[23] Essay AM Schenkelberg MA Von Seggern MJ Rosen MS Schlechter CR Rosenkranz RR. A protocol for a local community monitoring and feedback system for physical activity in organized group settings for children. J Phys Act Health. (2023) 20:385–93. doi: 10.1123/jpah.2022-0486 36965493 PMC10626975

[24] Edwards MB Theriault DS Shores KA Melton KM. Promoting youth physical activity in rural southern communities: practitioner perceptions of environmental opportunities and barriers. J Rural Health. (2014) 30:379–87. doi: 10.1111/jrh.12072, PMID: 24701977

[25] Wiggins DK. A worthwhile effort? History of organized youth sport in the United States. Kinesiol Rev. (2013) 2:65–75. doi: 10.1123/krj.2.1.65

[26] Frey JH Eitzen DS. Sport and society. Annu Rev Sociol. (1991) 17:503–22. doi: 10.1146/annurev.so.17.080191.002443

[27] Strand B Albrecht J. A review of the history of youth sports. (2010) 5:16–20.

[28] Logan K Cuff S. Council on sports medicine and fitness. Organized sports for children, preadolescents, and adolescents. Pediatrics. (2019) 143:1. doi: 10.1542/peds.2019-0997, PMID: 31110166

[29] Howie EK Daniels BT Guagliano JM. Promoting physical activity through youth sports programs: it’s social. Am J Lifestyle Med. (2020) 14:78–88. doi: 10.1177/1559827618754842, PMID: 31903087 PMC6933572

[30] Howie EK Guagliano JM Milton K Vella SA Gomersall SR Kolbe-Alexander TL. Ten research priorities related to youth sport, physical activity, and health. J Phys Act Health. (2020) 17:920–9. doi: 10.1123/jpah.2020-0151

[31] Lee JE Pope Z Gao Z. The role of youth sports in promoting children’s physical activity and preventing pediatric obesity: a systematic review. Behav Med. (2018) 44:62–76. doi: 10.1080/08964289.2016.1193462, PMID: 27337530

[32] Somerset S Hoare DJ. Barriers to voluntary participation in sport for children: a systematic review. BMC Pediatr. (2018) 18:47. doi: 10.1186/s12887-018-1014-1, PMID: 29426310 PMC5810190

[33] Hyde ET Omura JD Fulton JE Lee SM Piercy KL Carlson SA. Disparities in youth sports participation in the U.S., 2017-2018. Am J Prev Med. (2020) 59:e207–10. doi: 10.1016/j.amepre.2020.05.011, PMID: 32741540

[34] Kellstedt DK Schenkelberg MA Essay AM Von Seggern MJ Rosenkranz RR Welk GJ. Youth sport participation and physical activity in rural communities. Arch Public Health. (2021) 79:46. doi: 10.1186/s13690-021-00570-y, PMID: 33832548 PMC8028731

[35] Shull ER Dowda M Saunders RP McIver K Pate RR. Sport participation, physical activity and sedentary behavior in the transition from middle school to high school. J Sci Med Sport. (2020) 23:385–9. doi: 10.1016/j.jsams.2019.10.017, PMID: 31722841 PMC7054172

[36] Pfeiffer KA Dowda M Dishman RK McIver KL Sirard JR Ward DS. Sport participation and physical activity in adolescent females across a four-year period. J Adolesc Health. (2006) 39:523–9. doi: 10.1016/j.jadohealth.2006.03.005, PMID: 16982387

[37] Katzmarzyk PT Malina RM. Contribution of organized sports participation to estimated daily energy expenditure in youth. Pediatr Exerc Sci. (1998) 10:378–86. doi: 10.1123/pes.10.4.378

[38] U.S. Department of Health and Human Services. The National Youth Sports Strategy. Washington, DC: U.S. Department of Health and Human Services (2019).

[39] Umstattd Meyer MR Moore JB Abildso C Edwards MB Gamble A Baskin ML. Rural active living: a call to action. J Public Health Manag Pract. (2016) 22:E11–20. doi: 10.1097/PHH.0000000000000333, PMID: 26327514 PMC4775461

[40] Dzewaltowski DA. Whole-of-community systems intervention for youth population physical activity. Identifier: NCT03380143 (2017) Available at: https://clinicaltrials.gov/ct2/show/NCT03380143 (accessed June 25, 2020).

[41] Geverdt D Education demographic and geographic estimates program (EDGE): Locale boundaries file documentation, 2017 (NCES 2018–115). [Internet]. U.S. Department of Education. Washington, DC: National Center for Education Statistics (2019). Available at: http://nces.ed.gov/pubsearch (accessed December 14, 2020).

[42] National Center for Education Statistics. School & District Navigator [internet]. Common Core of Daa (CCD) school map. (2021). Available at: https://nces.ed.gov/ccd/schoolmap/ (accessed August 11, 2023).

[43] Welk GJ Saint-Maurice PF Dixon PM Hibbing PR Bai Y McLoughlin GM. Calibration of the online youth activity profile assessment for school-based applications. J Measure Phys Behav. (2021) 4:236–46. doi: 10.1123/jmpb.2020-0048 PMC10785831 38223785

[44] Welk GJ McLoughlin GM Lee JA Carrasco J. The utility of the youth activity profile for assessing and promoting physical activity in physical education. J Phys Educ Recreat Dance. (2023) 94:24–31. doi: 10.1080/07303084.2022.2136310

[45] Saint-Maurice PF Welk GJ. Web-based assessments of physical activity in youth: considerations for design and scale calibration. J Med Internet Res. (2014) 16:e269. doi: 10.2196/jmir.3626, PMID: 25448192 PMC4275492

[46] Saint-Maurice PF Bai Y Vazou S Welk G. Youth physical activity patterns during school and out-of-school time. Children (Basel). (2018) 5:118. doi: 10.3390/children5090118 30200255 PMC6162631

[47] Burns RD Bai Y Podlog LW Brusseau TA Welk GJ. Associations of physical activity enjoyment and physical education enjoyment with segmented daily physical activity in children: exploring tenets of the trans-contextual model of motivation. J Teach Phys Educ. (2022) 42:184–8. doi: 10.1123/jtpe.2021-0263

[48] Saint-Maurice PF Kim Y Hibbing P Oh AY Perna FM Welk GJ. Calibration and validation of the youth activity profile: the FLASHE study. Am J Prev Med. (2017) 52:880–7. doi: 10.1016/j.amepre.2016.12.010, PMID: 28526365 PMC5505319

[49] Data Resource Center for Child and Adolescent Health. Child and adolescent health measurement initiative: 2018-2019 National Survey of Children’s health (NSCH) data query [internet]. 2018-2019 National Survey of Children’s health (NSCH) data query. (2020) Available at: https://www.childhealthdata.org/ (Accessed February 22, 2022).

[50] Schenkelberg MA Essay AM Rosen MS Bavari AE Norgelas SJ Rosenkranz RR. A protocol for coordinating rural community stakeholders to implement whole-of-community youth physical activity surveillance through school systems. Prev Med Rep. (2021) 24:101536. doi: 10.1016/j.pmedr.2021.101536, PMID: 34976611 PMC8683876

[51] Harris PA Taylor R Thielke R Payne J Gonzalez N Conde JG. Research electronic data capture (REDCap): a metadata-driven methodology and workflow process for providing translational research informatics support. J Biomed Inform. (2009) 42:377–81. doi: 10.1016/j.jbi.2008.08.010, PMID: 18929686 PMC2700030

[52] Harris PA Taylor R Minor BL Elliott V Fernandez M O’Neal L. The REDCap consortium: building an international community of software platform partners. J Biomed Inform. (2019) 95:103208. doi: 10.1016/j.jbi.2019.103208, PMID: 31078660 PMC7254481

[53] Saint-Maurice PF Welk GJ. Validity and calibration of the youth activity profile. PLoS One. (2015) 10:e0143949. doi: 10.1371/journal.pone.0143949, PMID: 26630346 PMC4668067

[54] Flanagin A Frey T Christiansen SL. AMA manual of style committee. Updated guidance on the reporting of race and ethnicity in medical and science journals. JAMA. (2021) 326:621–7. doi: 10.1001/jama.2021.13304, PMID: 34402850

[55] Food and Nutrition Service, USDA. Child nutrition programs: income eligibility guidelines [internet]. Federal Register: The Daily Journal of the United States Government (2018). Available at: https://www.federalregister.gov/documents/2018/05/08/2018-09679/child-nutrition-programs-income-eligibility-guidelines (Accessed May 25, 2021).

[56] Harwell M LeBeau B. Student eligibility for a free lunch as an SES measure in education research. Educ Res. (2010) 39:120–31. doi: 10.3102/0013189X10362578

[57] Milliken GA Johnson DE. Analysis of Messy Data, Volume III: Analysis of Covariance. (*1st ed*.). Chapman and Hall/CRC (2001) 325–50.

[58] Littell RC Milliken GA Stroup WW Wolfinger RD Schabenberger O. SAS for mixed models, second edition. 2nd ed. Cary, N.C: SAS Institute (2006).

[59] Black LI Terlizzi EP Vahratian A. Organized sports participation among children aged 6-17 years: United States, 2020. NCHS Data Brief. (2022) 441:1–8. 35969661

[60] Aspen Institute project play. State of play 2022 [internet]. Aspen Institute (2022). Available at: https://www.aspenprojectplay.org/state-of-play-2022/participation-trends (Accessed February 18, 2023).

[61] Fleming DJM Dorsch TE Serang S Hardiman AL Blazo JA Farrey T. The association of families’ socioeconomic and demographic characteristics with parents’ perceived barriers to returning to youth sport following the COVID-19 pandemic. Psychol Sport Exerc. (2023) 65:102348. doi: 10.1016/j.psychsport.2022.102348, PMID: 36465329 PMC9710102

[62] Teare G Taks M. Exploring the impact of the COVID-19 pandemic on youth sport and physical activity participation trends. Sustain For. (2021) 13:1744. doi: 10.3390/su13041744

[63] Kelly AL Erickson K Turnnidge J. Youth sport in the time of COVID-19: considerations for researchers and practitioners. Manag Sport Leis. (2020) 27:62–72. doi: 10.1080/23750472.2020.1788975

[64] Johnson AM Bocarro JN Saelens BE. Youth sport participation by metropolitan status: 2018-2019 National Survey of Children’s health (NSCH). Res Q Exerc Sport. (2022) 94:895–04. doi: 10.1080/02701367.2022.2069662 35580038

[65] Johnston LD Delva J O’Malley PM. Sports participation and physical education in American secondary schools: current levels and racial/ethnic and socioeconomic disparities. Am J Prev Med. (2007) 33:S195–208. doi: 10.1016/j.amepre.2007.07.015 17884568

[66] Kwon S Letuchy EM Levy SM Janz KF. Youth sports participation is more important among females than males for predicting physical activity in early adulthood: Iowa bone development study. Int J Environ Res Public Health. (2021) 18:1328. doi: 10.3390/ijerph18031328, PMID: 33540518 PMC7908602

[67] Barker RG Gump PV. Big school, small school: high school size & student behavior. 1st ed. Stanford, CA: Stanford University Press (1964).

[68] Ferris KA Oosterhoff B Metzger A. Organized activity involvement among rural youth: gender differences in associations between activity type and developmental outcomes. J Res Rural Educ. (2013) 28:1–16. Available at: http://jrre.psu.edu/articles/28-15.pdf

[69] Baciu A. National Academies of Sciences, Engineering, and Medicine. Communities in Action: Pathways to Health Equity. Washington, DC: The National Academies Press (2017). 28418632

[70] Crane J Temple V. A systematic review of dropout from organized sport among children and youth. Eur Phys Educ Rev. (2015) 21:114–31. doi: 10.1177/1356336X14555294

[71] Waselewski A Waselewski M Waselewski E Kruger L Chang T. Perspectives of US youths on participation of transgender individuals in competitive sports: a qualitative study. JAMA Netw Open. (2023) 6:e2255107. doi: 10.1001/jamanetworkopen.2022.55107, PMID: 36753280 PMC9909496

[72] Jones BA Arcelus J Bouman WP Haycraft E. Sport and transgender people: a systematic review of the literature relating to sport participation and competitive sport policies. Sports Med. (2017) 47:701–16. doi: 10.1007/s40279-016-0621-y, PMID: 27699698 PMC5357259

[73] Ingram BJ Thomas CL. Transgender policy in sport, a review of current policy and commentary of the challenges of policy creation. Curr Sports Med Rep. (2019) 18:239–47. doi: 10.1249/JSR.0000000000000605, PMID: 31385840

[74] Pandya NK. Disparities in youth sports and barriers to participation. Curr Rev Musculoskelet Med. (2021) 14:441–6. doi: 10.1007/s12178-021-09716-5, PMID: 34622353 PMC8497066

[75] Wijtzes AI Jansen W Bouthoorn SH Pot N Hofman A Jaddoe VWV. Social inequalities in young children’s sports participation and outdoor play. Int J Behav Nutr Phys Act. (2014) 11:155. doi: 10.1186/s12966-014-0155-3 25510552 PMC4272790

[76] Brophy S Cooksey R Lyons RA Thomas NE Rodgers SE Gravenor MB. Parental factors associated with walking to school and participation in organised activities at age 5: analysis of the millennium cohort study. BMC Public Health. (2011) 11:14. doi: 10.1186/1471-2458-11-14 21210998 PMC3027134

[77] Nielsen G Hermansen B Bugge A Dencker M Andersen LB. Daily physical activity and sports participation among children from ethnic minorities in Denmark. Eur J Sport Sci. (2013) 13:321–31. doi: 10.1080/17461391.2011.635697, PMID: 23679149

[78] Zhang M Wang X-C Shao B. Predictors of persistent participation in youth sport: a systematic review and Meta-analysis. Front Psychol. (2022) 13:871936. doi: 10.3389/fpsyg.2022.871936 35712153 PMC9196305

[79] Malm C Jakobsson J Isaksson A. Physical activity and sports-real health benefits: a review with insight into the public health of Sweden. Sports (Basel). (2019) 7:127. doi: 10.3390/sports7050127 31126126 PMC6572041

[80] Tassitano RM Weaver RG Tenório MCM Brazendale K Beets MW. Physical activity and sedentary time of youth in structured settings: a systematic review and meta-analysis. Int J Behav Nutr Phys Act. (2020) 17:160. doi: 10.1186/s12966-020-01054-y, PMID: 33276782 PMC7716454

[81] Leek D Carlson JA Cain KL Henrichon S Rosenberg D Patrick K. Physical activity during youth sports practices. Arch Pediatr Adolesc Med. (2011) 165:294–9. doi: 10.1001/archpediatrics.2010.252 21135319

[82] Carlton T McKenzie TL Bocarro JN Edwards M Casper J Suau L. Objective assessment of physical activity and associated contexts during high school sport practices. Front Sports Act Living. (2021) 3:548516. doi: 10.3389/fspor.2021.548516 34308345 PMC8299060

[83] Whitt-Glover MC Taylor WC Floyd MF Yore MM Yancey AK Matthews CE. Disparities in physical activity and sedentary behaviors among US children and adolescents: prevalence, correlates, and intervention implications. J Public Health Policy. (2009) 30:S309–34. doi: 10.1057/jphp.2008.46, PMID: 19190581

[84] Nader PR Bradley RH Houts RM McRitchie SL O’Brien M. Moderate-to-vigorous physical activity from ages 9 to 15 years. JAMA. (2008) 300:295–305. doi: 10.1001/jama.300.3.295 18632544

[85] Trost SG Pate RR Sallis JF Freedson PS Taylor WC Dowda M. Age and gender differences in objectively measured physical activity in youth. Med Sci Sports Exerc. (2002) 34:350–5. doi: 10.1097/00005768-200202000-00025 11828247

[86] Gortmaker SL Lee R Cradock AL. Disparities in youth physical activity in the United States: 2003-2006. Med Sci Sports. (2012) 44:888–893. doi: 10.1249/MSS.0b013e31823fb254 22089478

[87] Pate RR Saunders RP Taverno Ross SE Dowda M. Patterns of age-related change in physical activity during the transition from elementary to high school. Prev Med Rep. (2022) 26:101712. doi: 10.1016/j.pmedr.2022.101712, PMID: 35145840 PMC8819127

[88] Farooq A Martin A Janssen X Wilson MG Gibson A-M Hughes A. Longitudinal changes in moderate-to-vigorous-intensity physical activity in children and adolescents: a systematic review and meta-analysis. Obes Rev. (2020) 21:e12953. doi: 10.1111/obr.12953, PMID: 31646739 PMC6916562

[89] Telford RM Telford RD Cochrane T Cunningham RB Olive LS Davey R. The influence of sport club participation on physical activity, fitness and body fat during childhood and adolescence: the LOOK longitudinal study. J Sci Med Sport. (2016) 19:400–6. doi: 10.1016/j.jsams.2015.04.008, PMID: 26111721

[90] Thompson HR London RA. Not all fun and games: disparities in school recess persist, and must be addressed. Prev Med Rep. (2023) 35:102301. doi: 10.1016/j.pmedr.2023.102301, PMID: 37408995 PMC10319329

[91] Stoepker P Dzewaltowski DA. Data coaching: a strategy to address youth physical behavior, motor competence, and out-of-school time leader evidence-based practices. J Phys Act Health. (2023):1–3. doi: 10.1123/jpah.2023-0673, PMID: [Epub ahead of print]. 38086365

[92] Gordon-Larsen P Adair LS Popkin BM. Ethnic differences in physical activity and inactivity patterns and overweight status. Obes Res. (2002) 10:141–9. doi: 10.1038/oby.2002.23 11886936

[93] Domogalla B Ko LK Jones R Ali WB Rodriguez E Duggan C. Rural Latino parent and child physical activity patterns: family environment matters. BMC Public Health. (2021) 21:2043. doi: 10.1186/s12889-021-12085-w, PMID: 34749683 PMC8577017

[94] Voss LD Hosking J Metcalf BS Jeffery AN Wilkin TJ. Children from low-income families have less access to sports facilities, but are no less physically active: cross-sectional study (EarlyBird 35). Child Care Health Dev. (2008) 34:470–4. doi: 10.1111/j.1365-2214.2008.00827.x, PMID: 18485026

[95] Cottrell L Zatezalo J Bonasso A Lattin J Shawley S Murphy E. The relationship between children’s physical activity and family income in rural settings: a cross-sectional study. Prev Med Rep. (2015) 2:99–04. doi: 10.1016/j.pmedr.2015.01.008 26844057 PMC4721359

[96] Sharp EH Tucker CJ Baril ME Van Gundy KT Rebellon CJ. Breadth of participation in organized and unstructured leisure activities over time and rural adolescents’ functioning. J Youth Adolesc. (2015) 44:62–76. doi: 10.1007/s10964-014-0153-4, PMID: 25037909

[97] Ludden AB. Engagement in school and community civic activities among rural adolescents. J Youth Adolesc. (2011) 40:1254–70. doi: 10.1007/s10964-010-9536-3, PMID: 20405186

[98] Krieger N Williams DR Moss NE. Measuring social class in US public health research: concepts, methodologies, and guidelines. Annu Rev Public Health. (1997) 18:341–78. doi: 10.1146/annurev.publhealth.18.1.341 9143723

[99] McGovern J. The intersection of class, race, gender and generation in shaping Latinas’ sport experiences. Sociol Spectr. (2021) 41:96–14. doi: 10.1080/02732173.2020.1850378

[100] Barr-Anderson DJ Flynn JI Dowda M Taverno Ross SE Schenkelberg MA Reid LA. The modifying effects of race/ethnicity and socioeconomic status on the change in physical activity from elementary to middle school. J Adolesc Health. (2017) 61:562–70. doi: 10.1016/j.jadohealth.2017.05.007, PMID: 28732715 PMC5654669

[101] Hasson RE. Addressing racial/ethnic differences in age-related declines in physical activity during adolescence. J Adolesc Health. (2017) 61:539–40. doi: 10.1016/j.jadohealth.2017.08.019, PMID: 29061230

[102] Garcia AS Takahashi S Anderson-Knott M Dev D. Determinants of physical activity for Latino and white middle school-aged children. J Sch Health. (2019) 89:3–10. doi: 10.1111/josh.12706, PMID: 30506697

[103] Appelqvist-Schmidlechner K Kyröläinen H Häkkinen A Vasankari T Mäntysaari M Honkanen T. Childhood sports participation is associated with health-related quality of life in young men: a retrospective cross-sectional study. Front Sports Act Living. (2021) 3:642993. doi: 10.3389/fspor.2021.642993, PMID: 33969295 PMC8100196

